# Motor Organization in Schizencephaly: Outcomes of Transcranial Magnetic Stimulation and Diffusion Tensor Imaging of Motor Tract Projections Correlate with the Different Domains of Hand Function

**DOI:** 10.1155/2021/9956609

**Published:** 2021-09-06

**Authors:** Ju-Yul Yoon, Da-Sol Kim, Gi-Wook Kim, Myoung-Hwan Ko, Jeong-Hwan Seo, Yu Hui Won, Sung-Hee Park

**Affiliations:** ^1^Department of Physical Medicine and Rehabilitation, Jeonbuk National University Medical School, Jeonju, Republic of Korea; ^2^Research Institute of Clinical Medicine of Jeonbuk National University-Biomedical Research Institute of Jeonbuk National University Hospital, Republic of Korea

## Abstract

**Objective:**

Schizencephaly is a rare congenital malformation that causes motor impairment. To determine the treatment strategy, each domain of the motor functions should be appropriately evaluated. We correlated a color map of diffusion tensor imaging (DTI) and transcranial magnetic stimulation (TMS) with the hand function test (HFT) to identify the type of hand function that each test (DTI and TMS) reflects. Further, we attempted to demonstrate the motor neuron organization in schizencephaly.

**Method:**

This retrospective study was conducted on 12 patients with schizencephaly. TMS was conducted in the first dorsal interosseous (FDI), biceps (BB), and deltoid muscles of the upper extremity, and contralateral MEP (cMEP) and ipsilateral MEP (iMEP) were recorded. The HFT included the grip strength, box and block (B&B), and 9-hole peg test. The schizencephalic cleft was confirmed using magnetic resonance imaging, and the corticospinal tract (CST) was identified using the color map of DTI. The symmetry indices for the peduncle and CST at pons level were calculated as the ratios of the cross-sectional area of the less-affected side and that of the more-affected side.

**Result:**

In the more-affected hemisphere TMS, no iMEP was obtained. In the less-affected hemisphere TMS, the iMEP response was detected in 9 patients and cMEP in all patients, which was similar to the pattern observed in unilateral lesion. Paretic hand grip strength was strongly correlated with the presence of iMEP (*p* = 0.044). The symmetry index of the color map of DTI was significantly correlated with the B&B (*p* = 0.008, *R*^2^ = 0.416), whereas the symmetry index of the peduncle was not correlated with all HFTs.

**Conclusion:**

In patients with schizencephaly, the iMEP response rate is correlated with the hand function related to strength, while the symmetricity of the CST by the color map of DTI is correlated with the hand function associated with dexterity. Additionally, we suggest the possible motor organization pattern of schizencephaly following interhemispheric competition.

## 1. Introduction

Schizencephaly is a congenital cerebral malformation characterized by the presence of one or more cerebral mantle clefts extending from the pial surface to the lateral ventricles and spanning the pial and ependymal surfaces of one or both cerebral hemispheres, delineated by the gray matter [1]. Schizencephaly is associated with several clinical features of varying severity, including developmental delay, intellectual disability, epilepsy, and motor deficits [2, 3].

“Cerebral palsy” (CP) diagnosis is based on the clinical symptoms rather than etiology, as a nonprogressive disorder occurring in the fetal brain during development which is expressed by permanent impairment of movement and posture [4]. Schizencephaly is one of the several brain lesions that lead to CP [5] but has distinct structural and temporal features compared to other brain lesions. There are three prominent features of schizencephaly, all of which are worth considering.

First, schizencephaly is a brain damage at an earlier stage than other lesions leading to CP and is not a 3rd trimester lesion, such as periventricular leukomalacia and stroke, but is a 2nd trimester lesion that has the highest plasticity [6]. Based on the abundant plasticity, the physiological manifestations in response to the brain damage can be better observed.

Second, the schizencephalic cleft and contralateral brain lesions are often concomitant. The bilateral schizencephalic cleft is the most common, and contralateral cortical dysgenesis often accompanies it [2]. Additionally, brain lesions, such as septo-optic dysplasia and agenesis of corpus callosum, tend to be frequently accompanied. These lesions appear to arise due to the developmental malformation of the telencephalic flexure [7]. Regarding bihemispheric brain lesion (schizencephalic cleft accompanying contralateral hemisphere lesion), Staudt mentioned that most previous CP studies have focused on unilateral brain lesions and exploring the differences that may appear in bilateral lesions would be helpful in understanding the organizational mechanism of the brain [8].

Third, motor manifestation is more common than the other deficits. According to a recent review of 734 patients with schizencephaly [2], motor deficits were the most common symptom and were observed in 90.3% of patients.

However, research on schizencephaly remains challenging for the following reasons: (1) low incidence rate (1.5/100,000 births in California), (2) various clinical presentations, (3) uncertain prognosis and unspecified treatment, and (4) limited studies with implemented evidence-based practice to understand schizencephaly [9].

Although not common, several studies have been conducted to understand schizencephaly. Structural abnormalities associated with schizencephaly include microcephaly, corpus callosum agenesis, septo-optic dysplasia, and cortical dysplasia [2]. Clinically related to these structural lesions, the bilateral schizencephalic clefts are less favorable for neurodevelopment (motor and cognition) than unilateral schizencephalic lesions [3]. In addition, the speech function was almost normal in patients with unilateral schizencephalic clefts but not those with bilateral schizencephalic clefts [3]. Neuronal migration disorders, such as cortical dysplasia, have more severe neurological symptoms [10]. According to recent studies, the most common symptoms of schizencephaly are motor and neurocognitive dysfunction. In particular, bilateral cleft, microcephaly, and corpus callosum agenesis are related to neurocognitive impairment [2]. In addition, Griffiths suggested the reclassification of schizencephaly and proposed the mechanism of cortical formation abnormality (predominantly, polymicrogyria) associated with schizencephalic cleft formation as an incomplete repair of destruction of the cortex that should have led to the schizencephalic cleft [11]. However, the research on schizencephaly is scarce and the pathophysiology and its underlying mechanism have not been clearly elucidated, although several attempts have been made.

The recent trend of research on developmental brain damage leading to CP not only has enabled the identification of the structural lesions of the cerebrum observed in magnetic resonance imaging (MRI) [12, 13] but also has taken advantage of the fact that it is possible to visualize the projection of the motor and sensory domains from the cortex to the spinal cord using diffusion tensor image (DTI). The visualization of the pathway could help people who were clinically diagnosed using MRI by extending the diagnosis to determine what type of progression could affect the areas of physiological function and facilitate the early determination of the future treatment direction [14–17].

In addition, transcranial magnetic stimulation (TMS) is an ideal tool to electrophysiologically examine the motor pathways in CP patients and is often used clinically for the diagnosis of central nervous system lesions [18]. The characteristics of the motor pathway can be distinguished by comparing the latency and amplitude of the ipsilateral and contralateral motor evoked potentials (MEP) [19]. Several previous studies of patients with CP have identified the ipsilateral motor pathway to be involved in motor pathway reorganization in response to damage [17, 20–23].

As mentioned, previous schizencephaly studies have focused on how function varies according to the accompanying concomitant lesions; however, to our best knowledge, no studies have attempted to evaluate motor function in patients with schizencephaly using MRI and DTI. In addition to finding structural concomitant lesions, functional variables could be measured through TMS and DTI tests. Furthermore, we attempted to explore findings that could be the basis for therapeutic application. The recent CP rehabilitation is divided into bimanual therapy and constraint-induced movement therapy (CIMT), and the measurement and evaluation of dysfunction as a therapeutic approach would serve as the basis for treatment selection [24]. In this study, we analyzed the TMS, DTI, and hand function test (HFT) in patients with schizencephaly and explored the clinical significance of the TMS and DTI tests in evaluating the motor function of schizencephaly.

## 2. Materials and Methods

### 2.1. Participants and Methods

#### 2.1.1. Participants

This study was a retrospective study; among the patients diagnosed with schizencephaly using MRI at the Jeonbuk National University Hospital between January 1, 2008, and December 31, 2019, those who received TMS were selected as participants in the study. The patient's age was not a limiting factor, and MRI was taken with DTI. As a result, we studied 12 patients with schizencephaly aged from 5 to 61 years (Table 1). This study was approved by the Institutional Review Board of Jeonbuk National University Hospital (approval number: CUH 2020-02-042), and all participants or their guardians provided written informed consent for their participation in this study.

#### 2.1.2. MRI

The Verio 3.0T system (Siemens, Erlangen, Germany) was used to obtain the MRI scans with DTI. The MRI system was equipped with a 12-channel head coil for single-shot echo planar imaging. The Generalized Autocalibrating Partially Parallel Acquisition (GRAPPA) technique, which is a part of the parallel imaging technique, was used. In addition, this technique produces better image quality due to the reduction of image distortion caused by the echoplanar imaging sequence. An image registration program built into the MRI system was used to correct for potential image distortion. The imaging parameters are as follows: echo time = 93 ms, repetition time = 7,900 ms, field of view = 230 mm × 230 mm, sampling matrix size = 128 × 128 reconstructed with homodyne processing to 256 × 256, sensitivity encoding (SENSE) factor = 3, echo planar imaging = 128, and *b* value = 1,000 s/mm^2^. A total of 47 contiguous, 3.0 mm thick slices were acquired parallel to the anterior commissure-posterior commissure line in 30 different diffusion directions. Anisotropy was calculated by using the orientation-independent fractional anisotropy (FA), and DTI color maps were created from FA values and the three vector elements. Vector maps were assigned color visualization, red (*x*, left-right), green (*y*, anteroposterior), and blue (*z*, superior-inferior) with a proportional intensity scale according to the FA. Visual assessment of white matter fibers on DTI color maps was performed. For MRI imaging equipment, quality control of several items, including geometric accuracy, high-contrast spatial resolution, and percent signal ghosting (artifact < 2.5%), was performed once a month and error in the three-dimensional position was minimized. In particular, since this study was based on the analysis of axial images, it was necessary to pay attention to the inclination or rotation of the section, which is the cause of such errors. During MRI scan preparation, the patients' heads were placed in the median position as much as possible, according to the technical procedure manual. Minor deviations such as head tilt were minimized by setting the axis through preliminary images.

#### 2.1.3. TMS

TMS was conducted using a MagPro (MagVenture, Lucernemarken, Denmark) equipped with a 7 cm, 8-shaped coil and the Medtronic Keypoint® electromyography workstation (Medtronic Inc., Skovlunde, Denmark); a 100 Hz high-pass filter and a 5 kHz low-pass filter were used.

C3 and C4 were identified according to the international 10–20 system. The recordings of ipsilateral motor evoked potentials (iMEPs) and contralateral motor evoked potentials (cMEPs) were made simultaneously at the bilateral first dorsal interosseous (FDI), biceps brachii (BB), and deltoid.

The threshold for the MEP parameters was set as the minimum stimulation intensity to obtain at least five out of 10 signals with a signal above 50 mV at a resting state. The optimal position of stimulation was the position where reproducible muscle response was obtained with minimal stimulation and was near C3 or C4. The baseline was kept as flat as possible, and MEPs obtained for threshold setting were discarded when the baseline was not flat [25]. The intensity of stimulation was set at 110% of the threshold. Four signals were obtained for each muscle; the average amplitude and the shortest latency were used for the analysis. When the MEP was not detected, even with the maximum output (100% of intensity) of the TMS device, it was set as “no response.” Stimuli were applied to each hemisphere in turn. Since cMEP and iMEP responses were obtained simultaneously, if no iMEP response was detected at the intensity that cMEP was measured (110% of threshold intensity), iMEP was defined as no response. A figure 8 coil (MC-B70) was used, and the maximum output at one stimulation was 1.45 tesla.

#### 2.1.4. HFT

Hand function was analyzed by the grip power test (grip), reflecting strength; the box and block (B&B) test, reflecting gross dexterity; and the 9-hole peg test (9-hole), reflecting fine dexterity [26]. All tests were conducted for both hands.

Grip power was evaluated as the unit of kilogram of force measured using a dynamometer for grip strength; the outcome for the B&B test was the number of times the block was moved to another box in 1 min; further, the outcome of the 9-hole was the time taken to remove and then reinsert nine pegs from a hole.

In the 9-hole, if the performance of the hand was so slow that it would take more than 600 s, the outcome of the test was taken to be 600 s to calculate the ratio of the paretic and less paretic hands [27].

### 2.2. Outcome Parameter

#### 2.2.1. Ratio of the Cross-Sectional Area of the DTI Color Map and Conventional MRI: The Symmetry Index

This study was targeted at patients with schizencephaly, and there are severe structural variabilities in each patient, such as the degree of cortical malformation and the size of the schizencephalic cleft. Therefore, it is difficult to capture the region of interest (ROI) at a structurally uniform location around the cortex.

Anatomically, representative locations where the motor tract passes (pons and peduncle in this study) are known from several studies [14, 28, 29]. Through the axial images of MRI and DTI at each of the abovementioned vertical positions (pons and peduncle), it was possible to discriminate the region reflecting the motor tract as a ROI. It could be inferred that the larger the size of the area reflecting the motor tract, the more active the motion information could be processed. Conversely, the smaller the size of the area, the less active the transmission of motion information would be [14, 29]. Therefore, we compared the ROI of the more-affected side and the less-affected side that reflect the motor tract and tried to determine which area of hand function it reflects.

Conventional brain MRI identified the schizencephalic cleft and concomitant lesions (Table 1). Using a T2-weighted fluid-attenuated inversion recovery (FLAIR) MRI, the area of the cerebral peduncles was measured in an axial plane, five or six sections below the anterior commissure-posterior commissure plane, passing through the mammillary bodies. The less-affected and more-affected areas were then compared to obtain the conventional MRI symmetry index [29] according to the following formula: the cross − sectional area of the more − affected side/cross − sectional area of the less − affected side.

In the DTI color map created from FA values and three vector elements, the corticofugal projection fibers were displayed in blue and the corticospinal tract (CST) was particularly well delineated at the level of the lower pons (level of the middle cerebellar peduncles), because all of the fibers run coherently in the craniocaudal direction [14, 30].

As a result, the cross-sectional area of the corticospinal tract (CST) at the pons level using the DTI color map (DTI-CST) and the cross-sectional area of the peduncle using the T2-FLAIR image (T2-peduncle) were identified and each symmetry index was obtained.

#### 2.2.2. DTI Tractography

In addition to the area-based analysis of the DTI color map, it was attempted to confirm the alignment of the CST fiber with a tractogram. The motor tract was reconstructed with a fiber tracking technology using DTI Studio software v.1.02 (CMRM, John Hopkins Medical Institute, Baltimore, MD, USA). Fiber tracking was performed with a fractional anisotropy (FA) threshold of 0.20 and a tract turning angle of 60.

To examine corticospinal dysgenesis, tracts were identified in the brain stem. The tractogram was created by assigning seed ROI in the upper pons (a portion of anterior blue color) and lower pons (a portion of anterior blue color; corresponds to DTI-CST) (Supplementary Figure 1) [31]. The fibers were visualized, and the average FA and ADC of the voxels composing the fiber were determined. The parameters were obtained for the more-paretic side and less-paretic side (Table 2).

#### 2.2.3. TMS Parameters: iMEP cMEP

In each of the more-affected and less-affected hemispheres, the response acquisition rate, amplitude, and latency of the cMEP and iMEP were analyzed.

In particular, we attempted to elucidate the role of iMEP that reflects the ipsilateral CST (iCST) in hand function. To confirm the function of iCST without contralateral CST (cCST) interference, we investigated the correlation between iMEP evocation status and hand function in patients who did not show cMEP corresponding to the paretic hand.

#### 2.2.4. HFT Parameters: The HFT Ratio

For the results of the grip and B&B tests and 9-hole measured for both the paretic and less-paretic sides, the ratio of the outcomes for the more-paretic hand and that for the less-paretic hand were used to quantitatively estimate the function of the paretic hand.

In the case of the grip and B&B tests, which use the amount of hand ability for a task as the outcome, the ratio was defined as “more paretic hand score/less paretic hand score.”

In the case of the 9-hole, which uses the time taken to perform a given task as the outcome, the ratio was defined as “less paretic hand completion time/more paretic hand completion time.”

Thus, the extent of decline in the function of the more paretic hand compared to the less paretic hand was quantitatively expressed (Table 3).

### 2.3. Statistical Analysis

The relationship between the symmetry index, which is the ratio of DTI-CST and T2-peduncle, and hand function, was analyzed using the Spearman correlation analysis. To measure the degree of correlation, the *R*^2^ value was measured. An *R*^2^ less than 0.2 indicated no correlation; 0.2 to 0.4, weak correlation; and greater than 0.4, correlation. After the Shapiro–Wilk normality test confirmed that the data did not satisfy normality, the Mann–Whitney *U* analysis was used to analyze the correlation between the ipsilateral MEP and hand function depending on the presence of evoked potential. The Wilcoxon rank sum test was used to compare the integrity (FA and ADC) of the less-affected CST and the more-affected CST in DTI-CST data. A *p* value < 0.05 was considered statistically significant.

## 3. Results

We recruited 12 patients with schizencephaly based on their MRI results and identified accompanying cerebral lesions (Table 1). Using the HFT, the more paretic hand was identified, and the results of the TMS and MRI were analyzed.

### 3.1. MEP Parameters

**Figure 1:**
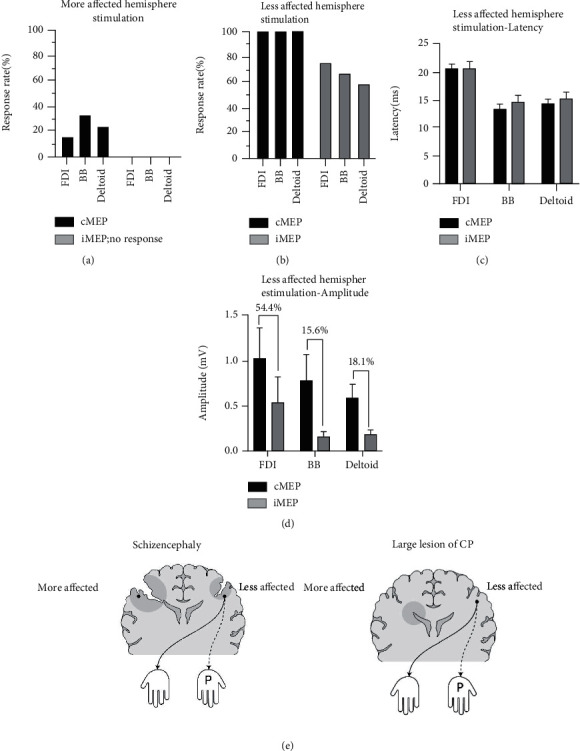
TMS results in schizencephaly patients and schematic depiction of the motor tract. (a) Motor evoked potential (MEP) response rate during stimulation of the more-affected hemisphere; no ipsilateral MEP (iMEP) response was detected, and the contralateral MEP (cMEP) response rate was very low rate. (b) MEP response rate during stimulation of the less-affected hemisphere; whereas cMEP was detected in all three muscles, iMEP response tended to dominate in the distal muscle (FDI). (c) Latency of iMEP and cMEP during stimulation of the less-affected hemisphere. (d) Amplitude of iMEP and cMEP during stimulation of the less-affected hemisphere; the number on the graph indicates the relative ratio of the amplitudes of iMEP and cMEP for each muscle, relatively larger in FDI. (e) Similarity of the motor organization pattern between schizencephaly and large lesion in cerebral palsy defined by Staudt; more affected hemispheres were unable to induce MEP by transcranial magnetic stimulation (TMS), and less-affected hemispheres induced MEP by TMS in both nonparalytic and paralytic hands.

The MEPs of the 12 patients with schizencephaly were obtained, and the iMEP and cMEP parameters were collected for each muscle. When the more-affected hemisphere was stimulated with TMS, none of the iMEP responses were obtained and 4 of 12 patients had cMEP responses in at least one of the FDI, BB, and deltoid muscles (*n* = 2, 4, and 3, respectively) (Figure 1(a), Supplementary Table 1). The four patients with cMEPs mentioned earlier had schizencephalic cleft and concomitant contralateral hemispheric lesions, such as polymicrogyria (patients 3, 6, 7, and 11; see Table 1). Of the four patients mentioned above, two had evoked potentials in all three muscles (FDI, BB, and deltoid; patients 6 and 11; see Table 1 and Supplementary Table 1).

On stimulating the less-affected hemisphere using TMS, cMEP responses were identified in all patients and iMEP responses were identified in 9 out of 12 patients, with different frequencies of iMEP response acquisition, depending on whether it was the proximal or distal muscle that was affected. Regarding the response acquisition rate of iMEP in the less-affected hemisphere, the FDI was most frequently evoked (9 of 12), followed by the BB (8 of 12), and then the deltoid muscle (7 of 12) (Figure 1(b); see iMEP, Supplementary Table 2). The latency of cMEP and iMEP in the less-affected hemisphere was virtually identical in all 3 muscles (Figure 1(c)), and the amplitude of iMEP was smaller than that of cMEP (Figure 1(d)).

**Figure 2:**
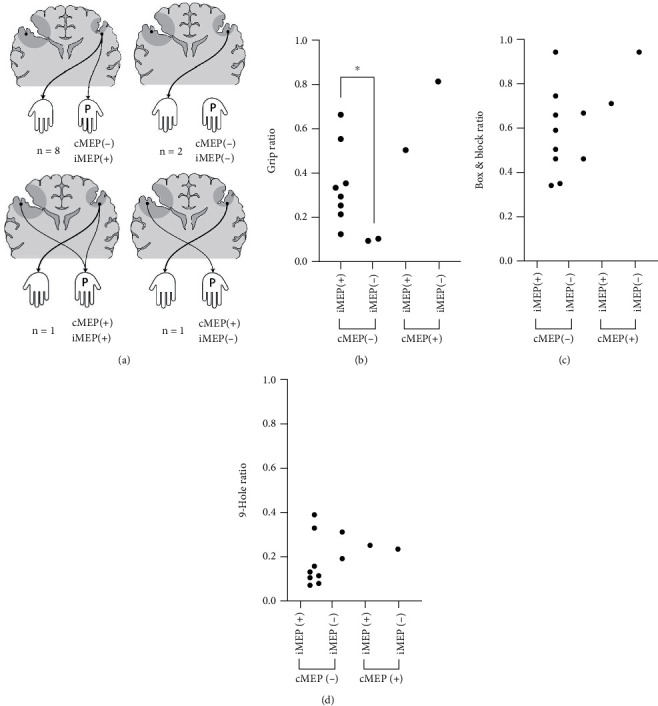
Schematic depiction of the MEP pattern towards the more paretic hand and the correlation between each pattern and hand function test. (a) Schematic depiction of four MEP patterns directed toward the paralyzed hand; the upper two patterns correspond to “large lesions” without cMEP response [22]. The presence or absence of the described cMEP and iMEP was presented as a variable in (b–d). (b) Grip results according to the presence or absence of iMEP and cMEP response; in the absence of cMEP reaction, the relationship between the presence of the ipsilateral tract and the results of the grip strength test; significant correlation is shown (*p* = 0.044). (c) B&B results according to the presence or absence of iMEP and cMEP responses; no significant difference was observed. (d) 9-hole results according to the presence or absence of iMEP and cMEP responses; no significant difference was observed. Grip: grip strength test; B&B: box and block test; 9-hole: 9-hole peg test.

Regarding the paretic hand, the motor signal supply occurs through two routes (contralateral wiring of the more-affected hemisphere or ipsilateral wiring of the less-affected hemisphere); to confirm the properties of the iMEP associated with the paretic hand and its role, it was necessary to exclude the cMEP from the more-affected hemisphere, which connects with the paretic hand. Patients classified in this way can be regarded as patients with a “large lesion,” based on the classification proposed by Staudt et al. [22] using MEP examination (Figure 1(e)). On stimulating the more-affected hemisphere, of the 12 patients, 10 obtained no cMEP response in the FDI and these 10 patients were analyzed for iMEP in the less-affected hemisphere. Of the 10 patients classified to have a “large lesion” according to the TMS result of the more-affected hemisphere, eight patients obtained iMEP response on stimulating the less-affected hemisphere and two did not (Figure 2(a); upper two schematics). When examining the relationship between the HFT results (grip, B&B, and 9-hole) and the presence of iMEP in the less-affected hemisphere for these 10 patients, the results of the grip test were significantly associated with the presence of iMEP (Figure 2(b); *p* = 0.044) but the results of the B&B test and 9-hole were not (Figures 2(c) and 2(d); *p* = 0.667 and *p* = 0.400, respectively).

### 3.2. MRI Findings in Schizencephaly

**Figure 3:**
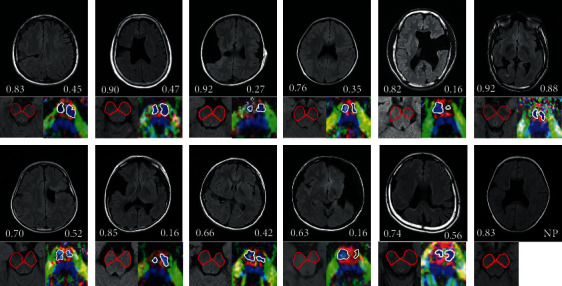
Conventional magnetic resonance imaging of schizencephaly patients and cross-sectional comparison of cerebral peduncle and corticospinal tract fibers in the pons. T2-fluid-attenuated inversion recovery (FLAIR) images of each schizencephaly patient (upper panel). Measurement of the cross-sectional area at the peduncle (lower red line) is shown in the lower left panel of each image, and the T2-peduncle symmetric index is inscribed above it. Measurement of the cross-sectional area of the corticospinal tract at the pons (lower white line) is shown in the lower right panel of each image, and the DTI-CST symmetric index is inscribed above it. NP: not performed.

MRI was performed for 12 patients, and T2-FLAIR images were obtained. DTI analysis was not performed for one patient, due to technical problems with DTI imaging; therefore, DTIs from the remaining 11 patients were analyzed (Figure 3). Of the 12 patients, 10 were detected to have bilateral hemispheric lesions, of which three lesions were bilateral schizencephalic clefts. Other schizencephaly-related contralateral lesions, such as polymicrogyria, which were observed, are specified in Table 1.

Overall, the symmetry index of DTI-CST tended to be smaller than that of T2-peduncle, which indicates that the area difference between the two sides was larger (Figure 3). In the tractography obtained using DTI studio (CMRM, John Hopkins Medical Institute, Baltimore, MD, USA), the average ADC value of voxel composing CST was larger in the more-affected side and the FA value of voxel composing CST was smaller in the more-affected side [32] (Table 2).

**Figure 4:**
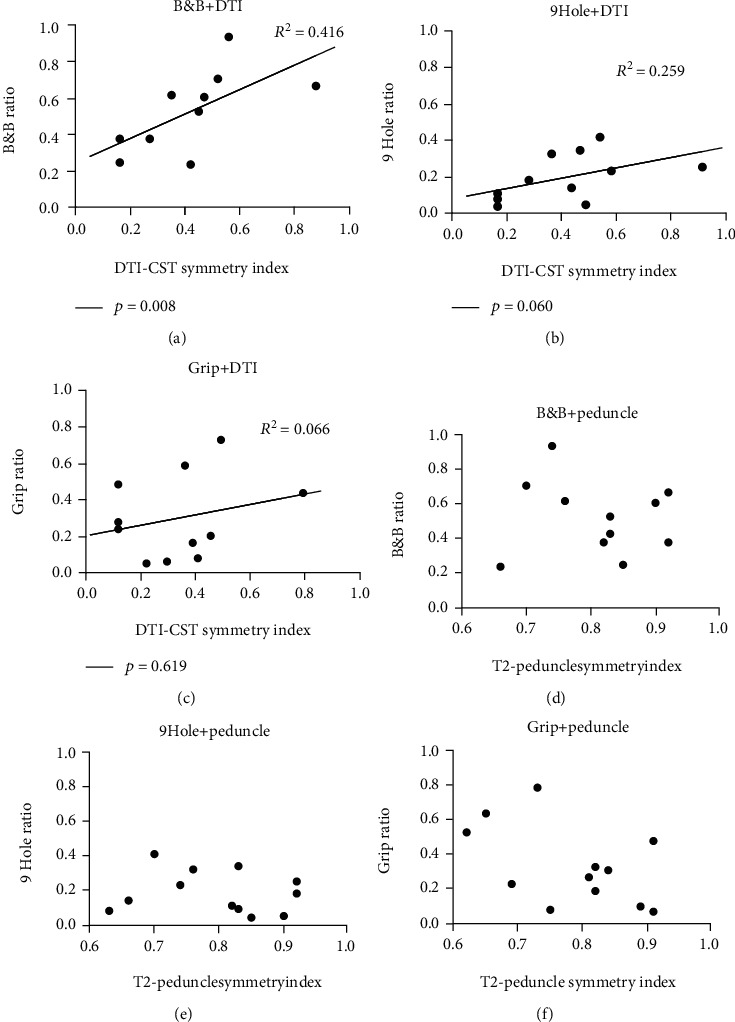
Correlation of the symmetry index and hand function test. (a) The symmetry index of DTI-CST and peduncle was correlated with the ratio of hand function evaluation. Box and block test (B&B) results and DTI-CST symmetry index show a significant correlation (*p* = 0.008, *R*^2^ = 0.416). (b) 9-hole peg test (9-hole) results and DTI-CST symmetry index showed a moderate correlation (*p* = 0.060, *R*^2^ = 0.259). (c) Grip strength test (grip) results did not show a significant correlation with the DTI-CST symmetry index (*p* = 0.619, *R*^2^ = 0.066). (d–f) The symmetry index of the peduncle did not show a significant correlation with hand function evaluation (B&B, *p* = 0.873 for (d); 9-hole, *p* = 0.649 for (e); and grip, *p* = 0.159 for (f)).

Regarding the symmetry index of DTI-CST, B&B was significantly correlated (*p* = 0.008, *R*^2^ = 0.416) and 9-hole displayed a tendency to be correlated (*p* = 0.060, *R*^2^ = 0.259). Alternatively, there was no correlation with the results of the grip test (*p* = 0.619, *R*^2^ = 0.066) (Figures 4(a)–4(c)). There was no significant correlation between the symmetric index of the peduncle and hand function (B&B *p* = 0.873, 9-hole *p* = 0.649, and grip *p* = 0.159) (Figures 4(d)–4(f)).

## 4. Discussion

In this study, we analyzed the correlation between electrophysiological (TMS) and radiological (DTI) tests and hand function tests in patients with schizencephaly. Our key findings are as follows. First, bilateral hemisphere lesions (schizencephalic cleft and concomitant contralateral lesion) were prevalent in schizencephaly (9 of 12) and the more-affected side was the hemisphere with a schizencephalic cleft, except for the bilateral schizencephalic cleft (Table 1). Second, on TMS, there was usually no MEP response in the more-affected side but the MEP response was detected in 4 out of 12 patients on the more-affected side (Table 1, Supplementary Table 1). Interestingly, all 4 patients had contralateral hemisphere lesions. Third, the presence of the iMEP response in the less-affected hemisphere demonstrated a significant increase in grip strength (Figure 2). Fourth, on the color-coded DTI map, comparison of the cross-sectional area of the bilateral motor tract in the section of pons revealed a relative decrease in the volume of the schizencephaly cleft side (Figure 3). In addition, a smaller area of DTI-CST on the more-affected side compared to that on the less-affected side was associated with poorer dexterity in the paralyzed hand (Figures 4(a) and 4(b); B&B and 9-hole, respectively). Our analyses permitted us to estimate the function of different territories that DTI and TMS tests each better reflect in patients with schizencephaly. Further, we have put forth a hypothesis to explain the motor organization pattern in fetal and neonatal brain damage.

### 4.1. Correlation of iMEP in the Less-Affected Hemisphere and Hand Function

Although most of the patients with schizencephaly in this study had bilateral hemisphere lesions, the response acquisition rate of iMEP as well as cMEP was much higher in the less-affected hemisphere than in the more-affected hemisphere (Figures 1(a) and 1(b)). This MEP pattern in schizencephaly patients is similar to that of “large lesions” in unilateral CP patients according to the criteria proposed by Staudt et al., who divided CP lesions into large, intermediate, and mild [22] (Figure 1(e)). In cerebral palsy, “large lesion” refers to a condition in which there is no MEP response upon stimulation of the more-affected hemisphere and MEP responses of both the paretic and nonparetic hands are obtained upon stimulation of the less-affected hemisphere [22].

It is noteworthy that the less-affected side with a concomitant contralateral lesion acts like the “intact side” of large lesion in unilateral CP, even though it is not in a normal state. When comparing the degree of motor function, the side with the schizencephalic cleft displayed appreciably more significant functional decline than the contralateral side in this study. This functional imbalance may lead to a monopoly of function into a relatively more functional hemisphere, such as the tilt of a balance scale. This is consistent with the concept of “interhemispheric inhibition,” which is discussed in a later section (Section 4.3).

From the more paretic hand's point of view, iCST is an incomplete but only channel that transmits stimulation, so it is necessary to consider which hand function iCST would affect. In the less-affected hemisphere, positive or negative response of iMEP response did not show a significant correlation in the HFTs reflecting dexterity (B&B test and 9-hole); however, the grip strength test results showed a significant increase in grip power when the positive response of iMEP was detected (Figure 2(b)). iMEP tracks iCST and cMEP tracks cCST [33]; therefore, iCST could be seen as a path of grip strength.

Even in other brain lesions, it has often been reported that iMEP reflects grip strength. A study on hemidisconnection (e.g., hemispherectomies and hemispherotomies) also reported that ipsilateral tracts (iCST) from the sound hemisphere existed in patients whose contralateral grasping ability of the removed hemisphere was preserved [34]. Similar to that observed in the unilateral CP, this ipsilateral projection would be able to rescue the motor function of the paretic hand [8, 35–37]. Other studies have demonstrated that unwithdrawn iCST could inhibit the normal development of cCST by continuously transmitting signal from the cortex, resulting in negative effects on the normal maturation of hand function [38–41]. However, this study differs in that only schizencephaly patients—reflecting early phase brain damage (late 1st or 2nd semester of pregnancy)—were assessed rather than all of the CP patient groups as in the previous studies. Moreover, in previous studies, the earlier the brain damage during pregnancy, the better the efficiency of the ipsilateral tract with respect to the hand function [6]. Therefore, schizencephaly could be considered to have a relatively more effective ipsilateral tract.

In the case of the B&B test and 9-hole, which are evaluations of dexterity, reflecting the ability to open hands and the time it takes to release, various factors are expected to intervene, not only iCST. Manual dexterity requires reciprocal inhibition of the flexor muscles since finger and wrist extensions are included, and a complex inhibitory mechanism acts during the coordination of hand movement. In the presence of brain lesions, dexterity could be significantly affected by neural manifestations, such as spasticity and hyperreflexia [42]. Abundant research indicates that several complex factors contribute to hand dexterity, including the ipsilateral secondary motor area [43], intercalated neurons (e.g., propriospinal neurons and reticulospinal neurons) [44], other regions within the cerebrum (i.e., intracortical inhibition and interhemispheric inhibition) [12, 45], parietal lobe, and subnetworks [46, 47]. In particular, studies of sensory organization on hand function were actively conducted [15, 48–51], and it was revealed that a phenomenon called “interhemispheric dissociation” which means the discrepancy between the organization of motor and sensory would have affected hand function [15, 29, 32, 40, 48, 52]. In summary, iCST could act as one contributing factor of dexterity, but there are several other contributing factors that act in a complex combination, so the effect of iCT on dexterity seems to be negligible.

### 4.2. Correlation between the Symmetry Index and Hand Function

In this study, the symmetry index is the ratio of the cross-sectional area of bilateral ROI [39]. The difference in the cross-sectional area could be caused by a variety of mechanisms, depending on how far the CST degeneration in the more-affected hemisphere has progressed or how the CST in the less-affected hemisphere has enhanced [32, 37–39, 49, 53].

On correlating the HFT results and DTI-CST symmetry index, DTI-CST symmetry index was correlated with dexterity (B&B test and 9-hole results), but not with grip strength test results (Figures 4(a)–4(c)). As mentioned in the previous section, dexterity is complicatedly influenced by several factors. If it is considered that the pons level CST is formed as a result of processing and synthesis of a series of processes in which the influencing factors act independently or interact, there may be a relationship between DTI-CST and hand function including dexterity. Moreover, along with the reduction in the cross-sectional area of the color-coded map, the DTI tractogram also showed a decrease in FA and ADC values (Table 2) [28, 32, 34, 49, 54].

However, in the case of the T2-peduncle, there was little correlation among all items of hand function. DTI is not usually performed in medical institutions because it requires an additional process and special equipment. Therefore, if the analysis of conventional MRI (T2-peduncle in this study) can identify hand function, it will be able to provide an advantage that can be applied to a wider range of patients (who did not have DTI) [55]. However, overall, the symmetry index of the T2-peduncle was greater than that of the DTI-CST, indicating that the less-affected and more-affected sides were measured to be less different than those of the DTI-CST (Figure 3). Since the DTI colormap visualizes the fiber itself and the T2-peduncle only sets the anatomical position, it seems that elements other than motor related are included in the area of the T2-peduncle [56]. Therefore, since the composition ratio of CST of T2-peduncle might be smaller than that of DTI-CST, it reflects motor function-related changes less sensitively than DTI-CST, and consequently, unlike DTI-CST, T2-peduncle does not significantly correlate with HFT.

**Figure 5:**
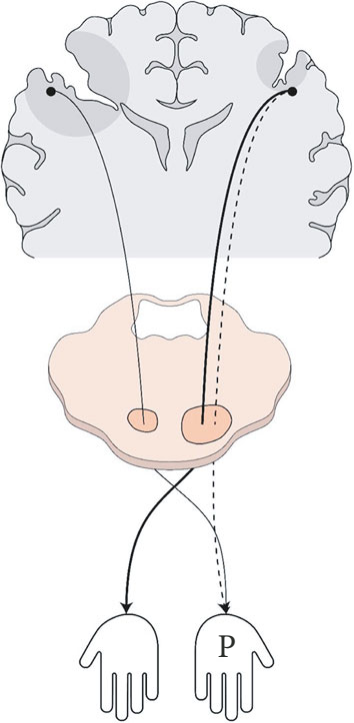
Schematic diagram of corticospinal tract projection in the less-affected hemisphere. Ipsilateral corticospinal tract (iCST) wiring related to the paretic hand (dotted line) was observed on the less-affected side of the pons before decussation of the lower medulla. Due to this iCST projection, the DTI-CST of the less-affected hemisphere cannot reflect purely the less-paretic hand and the DTI-CST of the more-affected hemisphere cannot reflect purely the more-paretic hand.

Interestingly, grip strength was not significantly correlated with DTI-CST in this study. This result should be considered in relation to the results of the correlation between the iMEP and grip strength analyzed earlier (with iMEP, the paretic hand grip power is stronger than that without iMEP) (Figure 2). A portion of the iCST was included in the DTI-CST area of the less-affected hemisphere, and this would act as an interference in the analysis of the correlation between the hand function and the DTI symmetry index, which indicates the quantitative relationship between the bilateral cross-sectional areas of the CST (Figure 5). That is, in the DTI-CST of the less-affected hemisphere, the portion responsible for the less paretic hand function (cCST) and the portion responsible for the more paretic hand function (iCST) are both included [31, 33](Figure 5). Since iMEP reflecting iCST is correlated with hand function (Figure 2(b); grip strength), DTI-CST observed on the less-affected side does not only reflect nonparetic hand function but also reflects paretic hand function (grip strength). Therefore, the correlation between the results of the grip strength test and the DTI-CST symmetry index would have been insignificant.

Similarly, in previous studies on patients with CP, DTI-CST was significantly correlated with hand function reflecting dexterity (e.g., digital dexterity and manual dexterity) than other functions [14]. Taking these results together, in patients with schizencephaly, DTI-CST appears to reflect dexterity (e.g., dexterity and coordination), while the presence of iMEP reflects grip strength.

### 4.3. Interpretation of Motor Organization in Schizencephaly

#### 4.3.1. Interhemispheric Competition

Since the schizencephalic cleft often has a concomitant contralateral lesion, the motor organization pattern of the bilateral hemisphere lesion could be observed. Two types of the MEP pattern were observed, and under the premise that iMEP tracks iCST and cMEP tracks cCST [33], a possible hypothesis about the “interhemispheric competition of both hemispheres lesion” could be suggested.

**Figure 6:**
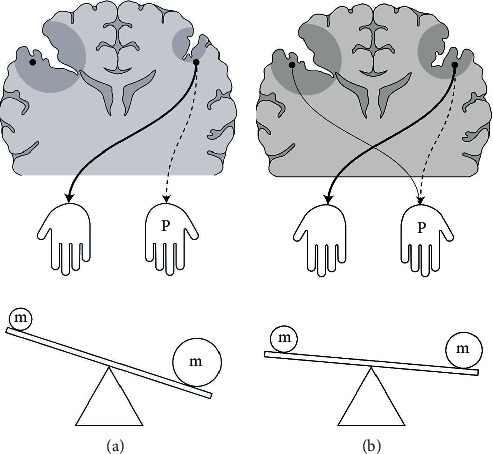
Schematic description of the interhemispheric competition of motor function in schizencephaly. Assuming that both (a) and (b) have the same degree of motor function invasion by the schizencephalic cleft in the right hemisphere, the activation of the contralateral corticospinal tract in the right hemisphere depends on the size of the concomitant lesion in the left hemisphere. The size of the circle marked “m” in the seesaw picture represents the degree of motor function. In (a), the weight of the motor function was inclined toward the left hemisphere, but in (b), the left and right hemispheres had similar weights of the motor function, and hence, they hardly tilted to one side.

First, for the pattern that no MEP (both cMEP and iMEP) response is obtained on the more-affected side (8 out of 12; Figure 6(a)), in this case, not only cMEP but also iMEP is often observed on the less-affected side. An interpretation of these results could be that an enhancement of the less-affected side activity is caused by the absence of inhibition from the more-affected side [45, 57] or that activity-dependent enhancement occurs due to continuous stimulation of the less-affected side to the paretic hand [35, 39]. Meanwhile, in the more-affected hemisphere, motor activity would be inactive and function would have been further degraded. Therefore, the unbalanced distribution of functions becomes more severe and the function becomes monopolized by one hemisphere.

Second, for the pattern that MEP evoked in the more-affected side, in some cases in this study, the degree of motor impairments of the less-affected hemisphere, a concomitant lesion, might be enough to compete with that of the more-affected hemisphere. In this condition, the less-affected side would not produce sufficient interhemispheric inhibition and an insufficient supply of motor signals to the hand would occur. Consequently, a contribution to motor function may occur not only in the less-affected hemisphere but also in the more-affected hemisphere (Figure 6(b)) [39]. In this study, there were 4 out of 12 patients whose cMEPs were measured by stimulation of the more-affected hemisphere (in at least one of the three muscles examined) (Figure 1(a), Supplementary Table 1), all of whom had lesions in the less-affected hemisphere (patients 3, 6, 7, and 11; Table 1, associated anomaly; three cortical dysplasias and one bilateral schizencephalic cleft). That is, even if the function of the more-affected hemisphere was severely reduced, the motor function would have been redistributed to the more-affected side, due to the lesion in the contralateral hemisphere.

Assuming the same degree of lesions in the more-affected hemisphere in these two conditions, we suggest that the more-affected hemisphere may acquire more motor function through competition if the degree of motor function deficit in the less-affected hemisphere is comparable to that of the more-affected side.

In summary, the motor organization in schizencephaly patients seems to reflect interhemispheric competition and we suggest that the motor organization pattern could vary according to the degree of influence of each lesion on the motor organization in both hemispheres [39, 58].

#### 4.3.2. Tendency to Withdrawal Failure of iCST in the Distal Upper Extremity

iMEP (9 out of 12 patients) and cMEP were evoked in the less-affected hemisphere (Figure 1(b)). Notably, the distal muscle (FDI) among the upper extremity muscles had a higher frequency and larger amplitude of iMEP response than the proximal muscles (BB, deltoid) (Figure 1(b), iMEP; Figure 1(d), iMEP). In addition, even in relative comparison of amplitude with the corresponding cMEP (iMEP/cMEP ratio) in each muscle, the ratio in FDI is larger than that of BB or deltoid (Supplementary Figure 2).

**Figure 7:**
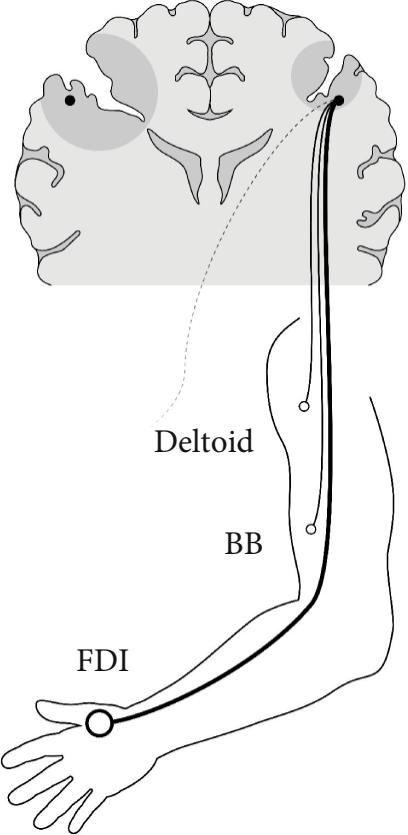
Schematic description of the dominance of iMEP according to muscle. iMEP tends to be more predominant in the distal muscle (FDI) than proximal muscles (BB, deltoid) in the upper extremity, which may be attributed to the tendency of iCST withdrawal failure in the distal muscle rather than in the proximal muscles. iMEP: ipsilateral motor evoked potential; FDI: first dorsal interosseous; BB: biceps brachii; iCST: ipsilateral corticospinal tract.

With regard to the projection and withdrawal of the normal iCST, several studies have mentioned that the ipsilateral tract, which is completed during fetal development, disappears around 2 years after birth [17, 59]. However, in pathologic conditions, such as fetal or perinatal brain damage, the ipsilateral tract is not withdrawn and is maintained [60]. Based on this information, our findings could be interpreted as a tendency to withdrawal failure of the ipsilateral tract in the distal upper extremity (Figure 7).

iMEP tends to be more dominant in the distal than proximal muscles in terms of the amplitude and frequency of evocation in this study. Similar results were obtained for iMEP in previous studies of patients with congenital mirror movement disorder (CMMD) which is caused by the focal expression of gene mutations related to abnormal pyramidal decussation [61–63]. The tendency of preservation of the ipsilateral tract to be predominant in distal muscle than proximal, even in two different disease groups, could be regarded as a distinct feature of the ipsilateral tract.

Factors that can be considered the cause of this phenomenon include the disturbance of withdrawal according to an activity-dependent mechanism [39, 60] due to more stimulation on the distal part (hand) than the proximal part of the upper extremities or the distribution of factors, such as axon guidance molecules and growth factors that influence neural organization [64]. In addition, it might be attributed to the tendency of functional migration of the hand region, which is greater than that of the other proximal upper limb regions, as language function migrates from the dominant hemisphere to the nondominant hemisphere in response to damage of the dominant hemisphere [65]. However, further research is warranted to fully understand this phenomenon.

In summary, we suggest that iMEP tends to be more predominant in the distal than the proximal part in the upper extremity, which seems to be due to the tendency of iCST withdrawal failure in the distal muscle rather than in the proximal muscles.

## 5. Future Research Direction

In this study, most patients with schizencephaly tended to have bilateral hemisphere lesions (schizencephalic cleft and concomitant contralateral lesion) and “interhemispheric competition” was suggested as a motor organization pattern. However, other factors may have an effect, and as mentioned above, one of the factors that greatly affects motor organization is the sensory area [40, 49]. If further studies on the organization of the sensory pathway for early brain damage with bilateral hemispheric lesions are conducted, more sophisticated correlations with motor functions could be expected; i.e., if the somatosensory evoked potential study and tensor image analysis reflecting the sensory tract are added and considered in addition to the methods used in this study, it is expected that a more in-depth understanding of the motor function will be possible by correlating their results with the trend of the ipsilateral or contralateral projection of the sensory tract, such as interhemispheric dissociation [40].

In addition, in this study, the ipsilateral motor tract displayed a dominant tendency in the distal muscle of the upper extremity, compared to proximal muscles. For the withdrawal failure of the ipsilateral tract that differs depending on the muscle, it seems necessary to study a larger number of subjects, incorporating the contralateral motor organization and its mechanism.

Grip strength and manual dexterity were correlated with the iMEP evocation status and the DTI-CST symmetry index of the less-affected hemisphere, respectively. Measurement of iMEP and the DTI-CST symmetry index before practical treatment could suggest the direction of treatment such as constraint-induced movement therapy and bimanual therapy, even therapeutic repetitive TMS or transcranial direct current stimulation [17, 24, 66–69]. This study could provide the basis for further studies that correlate TMS or DTI results with practical treatment.

## 6. Limitations

Even with schizencephaly, certain individuals may be asymptomatic [1, 70]. Since this study was conducted among a group of symptomatic patients, it is possible that only patients with high severity were assessed. The sample size used in this study was small, and as a study on a single disease group rather than a wide range of brain damage, it is insufficient to explain the motor organization of the entire CP. But considering the very rare incidence and the unique characteristics compared to other developmental brain damages, the analysis of schizencephaly remains meaningful.

In this study, the integrity and symmetricity of CST were measured using color-coded DTI analysis and this attempt had the advantage of being practical and intuitively applicable to clinical situations. Area-based analysis of color-coded DTI may cause errors. In particular, errors may occur depending on the inclination of the axis according to the angle or rotation of the section of the image. Moreover, DTI is very sensitive and some artifacts often occur due to eddy currents and motion [71]. In the MRI system, quality control was performed once a month to minimize errors and distortions, and in terms of imaging methods, errors due to axes or sections were minimized through preliminary imaging but it was not completely error free. In fact, for one patient (patient 12), the DTI acquired at the peduncle–stem level was lost and DTI analysis of the patient was not possible.

In addition, the integrity of iCST may vary with age among healthy people [72]. The iCST that appears in patients with schizencephaly may also have age-related differences; in this study, the age of the patients when DTI was performed was from 5 to 61 years and the analysis was conducted without dividing the data for children and adults. However, in the case of pathologic iCST of patients with CP, active ipsilateral projection tends not to withdraw and there should be no significant change in integrity with age [20]. Therefore, the age of schizencephaly patients would have little influence on the analysis of the motor organization pattern.

## 7. Conclusion

This study examined the aspects of motor organization in schizencephaly using TMS and DTI reflecting the early-stage embryological brain damage. Our results suggest that the ipsilateral motor tract, which is often observed in the less-affected hemisphere of patients with schizencephaly, has a greater influence on the hand function related to strength and that the cross-sectional area symmetricity of CST on pons is associated with the hand function related to dexterity. Further, motor organization following interhemispheric competition was observed in schizencephaly, which often presented as bilateral hemispheric lesions. We suggest that more variables may be involved in the organization of motor function and warrants a future study with a larger sample size.

## Figures and Tables

**Table 1:** Demographic data of patients with schizencephaly.

Case	Age	Sex	MRI findings		Motor	Cognition	Spasticity^5^	Associated symptom
			Type	Associated anomaly	MMT (upper lower)			
1	24	F	Rt F-T schizencephaly, closed lip	Lt F cortical dysplasia, absent septum pellucidum	N/G N/G	MMSE 30	None	
2	61	F	Rt F-T schizencephaly, open lip	Absent septum pellucidum	N/F N/F	SQ 62^1^	Lt 1	
3	11	F	Rt F-T schizencephaly, open lip	Lt polymicrogyria, Lt arachnoid cyst, septo-optic dysplasia, absent septum pellucidum	G/F G/F	IQ 43^2^, SQ 83^4^	Lt 1	Seizure
4	6	F	Lt F schizencephaly, closed lip	Rt F polymicrogyria	F/N F/N	IQ 95^3^, SQ 106^4^	Rt 1	
5	16	M	Lt F-T schizencephaly, open lip	Absent septum pellucidum	P/F P/F	IQ 61^2^, SQ 76^4^	Rt 1	
6	25	M	Rt T-P schizencephaly, closed lip	Lt F-T cortical dysplasia	G/P G/P	IQ 45^1^, SQ 37^4^	Rt 2, Lt 3	Seizure
7	9	M	Lt F-T schizencephaly, closed lip	Rt cortical dysplasia, absent septum pellucidum	G/N G/N	IQ 82^2^, SQ 93^4^	Rt 1	
8	8	F	Bilateral schizencephaly, Rt F-T open lip, Lt T-P closed lip	Absent septum pellucidum	G/P F/P	IQ 121^2^, SQ 110^4^	Lt 1	
9	15	F	Bilateral schizencephaly, both F-T closed lip	—	P/P P/P	IQ 43^2^, SQ 35^4^	Rt 2, Lt 1	
10	9	F	Lt F-T schizencephaly, open lip	—	F/N F/N	IQ 38^2^, SQ 25^4^	Rt 1	
11	41	M	Bilateral schizencephaly, both P closed lip	Rt F cortical dysplasia	F/F T/T	MMSE 15	Rt 3, Lt 3	
12	5	F	Rt schizencephaly, closed lip	—	N/G N/G	IQ 89^3^	Lt 1	

NP: not performed; NT: not testable; Rt: right; Lt: left; M: male; F: female; ^∗^F-T-P: fronto-temporo-parietal; IQ: intelligence quotient; SQ: social quotient; MMSE: mini-mental state examination; MMT: manual muscle testing; Dx: diagnosis. ^1^Wechsler Adult Intelligence Scale-III; ^2^Wechsler Intelligence Scale for Children-III; ^3^Wechsler Preschool and Primary Scale of Intelligence; ^4^Social Maturity Scale; ^5^Modified Ashworth Scale.

**Table 2:** CST characteristics in DTI.

	ADC (×10^−4^)			FA		
	More-affected CST	Less-affected CST	*p*	More-affected CST	Less-affected CST	*p*
*Mean* ± *SD*	8.38 ± 0.324	7.350 ± 0.459	0.005^∗^	0.514 ± 0.088	0.657 ± 0.040	0.013^∗^

Values are presented as the mean ± standard deviation. CST: corticospinal tract; DTI: diffusion tensor imaging; FA: fractional anisotropy; ADC: average apparent diffusion coefficient; SD: standard deviation. ^∗^*p* < 0.05.

**Table 3:** Hand function test results of patients with schizencephaly.

Case no.	More paretic hand	HFT ratio		
		Grip (kg; paretic : less paretic)	B&B (*n*; paretic : less paretic)	9-hole (sec; paretic : less paretic)
1	Lt	0.21 (7 : 34)	0.53 (31 : 58)	0.35 (46 : 16)
2	Lt	0.12 (2 : 17)	0.61 (25 : 41)	0.06 (408 : 23)
3	Lt	0.09 (0.5 : 5.5)	0.38 (8 : 21)	0.19 (NT : 93)
4	Rt	0.1 (0.4 : 4)	0.62 (18 : 29)	0.33 (79 : 26)
5	Rt	0.29 (4 : 14)	0.38 (15 : 40)	0.12 (173 : 20)
6	Lt	0.5 (9 : 18)	0.67 (14 : 21)	0.26 (140 : 37)
7	Rt	0.25 (1 : 4)	0.71 (37 : 52)	0.42 (66 : 28)
8	Lt	0.33 (4 : 12)	0.25 (10 : 40)	0.05 (NT : 23)
9	Rt	0.66 (4 : 6)	0.24 (6 : 25)	0.15 (NT : 76)
10	Rt	0.55 (2.2 : 4)	NP	0.09 (NT : 47)
11	Lt	0.81 (13 : 16)	0.94 (17 : 18)	0.24 (225 : 54)
12	Lt	0.35 (3 : 8.6)	0.43 (19 : 44)	0.10 (260 : 27)
*Mean* ± *SD*		0.355 ± 0.22	0.524 ± 0.20	0.196 ± 0.12

HFT: hand function test; B&B: box and block test; Grip: grip strength test; 9-hole: 9-hole peg test; Rt: right; Lt: left; kg: kilogram; *n*: number of blocks; sec: second; SD: standard deviation; NT: not testable; NP: not performed.

## Data Availability

The original contributions presented in the study are included in the article/supplementary material; further inquiries can be directed to the corresponding authors.

## Abbreviations

DTI: Diffusion tensor imaging
TMS: Transcranial magnetic stimulation
HFT: Hand function test
MEP: Motor evoked potential
FDI: First dorsal interosseous
BB: Biceps
cMEP: Contralateral MEP
iMEP: Ipsilateral MEP
B&B: Box and block
CST: Corticospinal tract
DTI-CST: DTI color map
ADC: Apparent diffusion coefficient
FA: Fractional anisotropy
FDI: First dorsal interosseous
CP: Cerebral palsy
MRI: Magnetic resonance imaging
FLAIR: Fluid-attenuated inversion recovery
9-hole: 9-hole peg test
Grip: Grip power test
CMMD: Congenital mirror movement disorder.

## Supplementary Materials

Supplementary Materials Supplementary Figure 1: the seed ROI setup for DTI tractogram of case 4 patient; the blue portion of the color DTI of the upper pons (ROI#1) and the lower pons (ROI#2; correspond to DTI-CST) is designated. Supplementary Figure 2: the relative comparison of iMEP amplitude with the corresponding cMEP amplitude; the ratio of iMEP amplitude/cMEP amplitude compared for each muscle. Supplementary Table 1 and Supplementary Table 2: the data of TMS for the more-affected and less-affected hemispheres.
