# Supplementary material for: Motor Organization in Schizencephaly: Outcomes of Transcranial Magnetic Stimulation and Diffusion Tensor Imaging of Motor Tract Projections Correlate with the Different Domains of Hand Function
Source: Biomed Res Int. 2021 Sep 6;2021:9956609. doi: 10.1155/2021/9956609 (PMC8437638; doi:10.1155/2021/9956609)
Supplement: Supplementary Materials — Supplementary Figure 1: the seed ROI setup for DTI tractogram of case 4 patient; the blue portion of the color DTI of the upper pons (ROI#1) and the lower pons (ROI#2; correspond to DTI-CST) is designated. Supplementary Figure 2: the relative comparison of iMEP amplitude with the corresponding cMEP amplitude; the ratio of iMEP amplitude/cMEP amplitude compared for each muscle. Supplementary Table 1 and Supplementary Table 2: the data of TMS for the more-affected and less-affected hemispheres. [file 9956609.f1.zip › Supplementary_figure_1_Revision.docx]

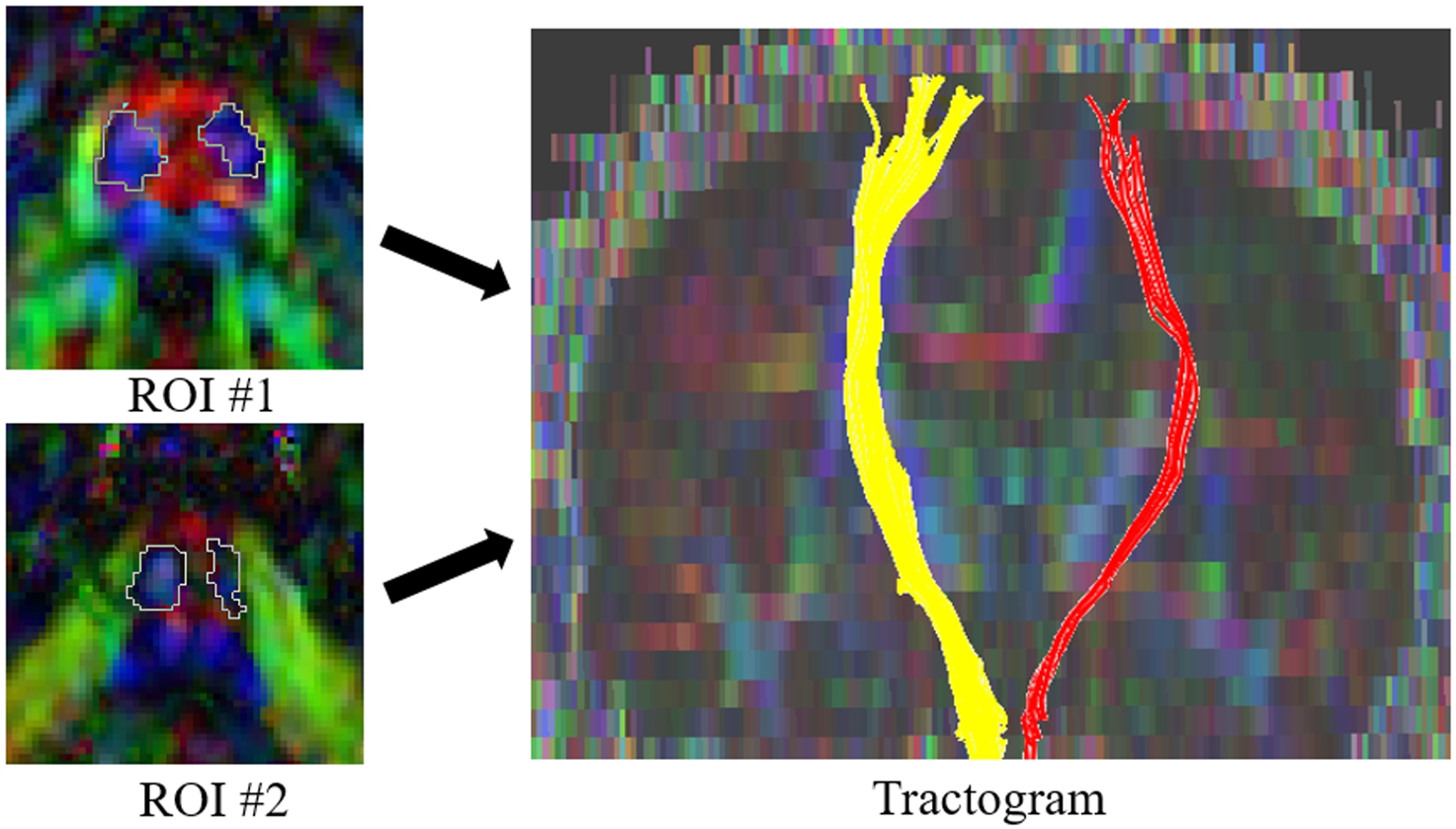


**Supplementary Figure 1**. Seed ROIs setup for DTI tractogram of Case 4 patient

The blue portion of the color DTI of the upper pons(ROI#1) and the lower pons (ROI#2;correspond to DTI-CST) was measured as ROI for tractography of CST. To check the intergrity of CST with a DTI tractogram, the average values of FA and ADC values of voxels constituting the fiber were obtained, and the less affected side and the more affected side were compared.
