# Supplementary material for: Motor Organization in Schizencephaly: Outcomes of Transcranial Magnetic Stimulation and Diffusion Tensor Imaging of Motor Tract Projections Correlate with the Different Domains of Hand Function
Source: Biomed Res Int. 2021 Sep 6;2021:9956609. doi: 10.1155/2021/9956609 (PMC8437638; doi:10.1155/2021/9956609)
Supplement: Supplementary Materials — Supplementary Figure 1: the seed ROI setup for DTI tractogram of case 4 patient; the blue portion of the color DTI of the upper pons (ROI#1) and the lower pons (ROI#2; correspond to DTI-CST) is designated. Supplementary Figure 2: the relative comparison of iMEP amplitude with the corresponding cMEP amplitude; the ratio of iMEP amplitude/cMEP amplitude compared for each muscle. Supplementary Table 1 and Supplementary Table 2: the data of TMS for the more-affected and less-affected hemispheres. [file 9956609.f1.zip › Supplementary_figure_2_Revision.docx]

**Supplementary Figure 2.** Relative comparison of iMEP amplitude with the corresponding cMEP amplitude

When the less-affected hemisphere is stimulated, the iMEP amplitude / cMEP amplitude is measured higher in FDI than in other muscles. This may be a phenomenon in which iMEP acts more predominantly in the distal muscle, which means relatively more withrawal failure than other proximal muscles.
