# Supplementary material for: Motor Organization in Schizencephaly: Outcomes of Transcranial Magnetic Stimulation and Diffusion Tensor Imaging of Motor Tract Projections Correlate with the Different Domains of Hand Function
Source: Biomed Res Int. 2021 Sep 6;2021:9956609. doi: 10.1155/2021/9956609 (PMC8437638; doi:10.1155/2021/9956609)
Supplement: Supplementary Materials — Supplementary Figure 1: the seed ROI setup for DTI tractogram of case 4 patient; the blue portion of the color DTI of the upper pons (ROI#1) and the lower pons (ROI#2; correspond to DTI-CST) is designated. Supplementary Figure 2: the relative comparison of iMEP amplitude with the corresponding cMEP amplitude; the ratio of iMEP amplitude/cMEP amplitude compared for each muscle. Supplementary Table 1 and Supplementary Table 2: the data of TMS for the more-affected and less-affected hemispheres. [file 9956609.f1.zip › Supplementary_table_1-Revision.docx]

**Supplementary Table 1.** Transcranial magnetic stimulation of the more affected hemisphere

| Case No | More affected hemisphere stimulation: Contralateral recording  (latency; msec / amplitude; mV / intensity; %) | | | More affected hemisphere stimulation: Ipsilateral recording  (latency; msec / amplitude; mV / intensity; %) | | |
| --- | --- | --- | --- | --- | --- | --- |
|  | FDI | BB | Deltoid | FDI | BB | Deltoid |
| 1 | - / - / 100 | - / - / 100 | - / - / 100 | - / - / 100 | - / - / 100 | - / - / 100 |
| 2 | - / - / 100 | - / - / 100 | - / - / 100 | - / - / 100 | - / - / 100 | - / - / 100 |
| 3 | - / - / 100 | 10.9 / 0.97 / 44 | - / - / 100 | - / - / 100 | - / - / 44 | - / - / 100 |
| 4 | - / - / 100 | - / - / 100 | - / - / 100 | - / - / 100 | - / - / 100 | - / - / 100 |
| 5 | - / - / 100 | - / - / 100 | - / - / 100 | - / - / 100 | - / - / 100 | - / - / 100 |
| 6 | 23.7 / 0.05 / 90 | 16.9 / 0.97 / 59 | 16.3 / 1.58 / 58 | - / - / 90 | - / - / 59 | - / - / 58 |
| 7 | - / - / 100 | 27.5 / 0.10 / 70 | 27.6 / 0.15 / 78 | - / - / 100 | - / - / 70 | - / - / 78 |
| 8 | - / - / 100 | - / - / 100 | - / - / 100 | - / - / 100 | - / - / 100 | - / - / 100 |
| 9 | - / - / 100 | - / - / 100 | - / - / 100 | - / - / 100 | - / - / 100 | - / - / 100 |
| 10 | - / - / 100 | - / - / 100 | - / - / 100 | - / - / 100 | - / - / 100 | - / - / 100 |
| 11 | 19.8 / 0.10 / 42 | 12.6 /0.41 / 48 | 11.5 / 0.21 / 50 | - / - / 42 | - / - / 48 | - / - / 50 |
| 12 | - / - / 100 | - / - / 100 | - / - / 100 | - / - / 100 | - / - / 100 | - / - / 100 |

FDI, first dorsal interosseous muscle; BB, biceps brachii muscle; msec, milliseconds; mV, millivolts; “ - “, not evoked.
