# Supplementary material for: Motor Organization in Schizencephaly: Outcomes of Transcranial Magnetic Stimulation and Diffusion Tensor Imaging of Motor Tract Projections Correlate with the Different Domains of Hand Function
Source: Biomed Res Int. 2021 Sep 6;2021:9956609. doi: 10.1155/2021/9956609 (PMC8437638; doi:10.1155/2021/9956609)
Supplement: Supplementary Materials — Supplementary Figure 1: the seed ROI setup for DTI tractogram of case 4 patient; the blue portion of the color DTI of the upper pons (ROI#1) and the lower pons (ROI#2; correspond to DTI-CST) is designated. Supplementary Figure 2: the relative comparison of iMEP amplitude with the corresponding cMEP amplitude; the ratio of iMEP amplitude/cMEP amplitude compared for each muscle. Supplementary Table 1 and Supplementary Table 2: the data of TMS for the more-affected and less-affected hemispheres. [file 9956609.f1.zip › Supplementary_table_2-Revision.docx]

**Supplementary Table 2.** Transcranial magnetic stimulation of the less affected hemisphere

| Case No | Less affected hemisphere stimulation: Contralateral recording  (latency; msec / amplitude; mV / intensity; %) | | | Less affected hemisphere stimulation: Ipsilateral recording  (latency; msec / amplitude; mV / intensity; %) | | |
| --- | --- | --- | --- | --- | --- | --- |
|  | FDI | BB | Deltoid | FDI | BB | Deltoid |
| 1 | 20.53 / 0.42 / 55 | 16.38 / 0.36 / 43 | 15.58 / 0.43 / 45 | 19.00 / 0.39 / 55 | 15.48 / 0.21 / 43 | 15.78 / 0.31 / 45 |
| 2 | 17.50 / 0.86 / 34 | 11.70 / 0.77 / 40 | 14.80 / 0.75 / 39 | 17.90 / 0.35 / 34 | 13.98 / 0.40 / 40 | 15.00 / 0.34 / 39 |
| 3 | 16.20 / 1.48 / 32 | 10.60 / 0.77 / 34 | 9.78 / 0.54 / 34 | - / - / 32 | - / - / 34 | - / - / 34 |
| 4 | 21.25 / 0.65 / 72 | 10.38 / 0.65 / 72 | 8.88 / 0.78 / 72 | - / - / 72 | - / - / 72 | - / - / 72 |
| 5 | 22.00 / 0.33 / 60 | 17.80 / 0.14 / 67 | 17.33 / 0.30 / 62 | 22.50 / 0.24 / 60 | 17.65 / 0.13 / 67 | 17.83 / 0.21 / 62 |
| 6 | 21.18 / 4.20 / 54 | 13.55 / 3.72 / 54 | 17.25 / 2.00 / 54 | 21.85 / 2.07 / 54 | - / - / 54 | - / - / 54 |
| 7 | 28.78 / 0.10 / 65 | 20.23 / 0.15 / 65 | 18.13 / 0.23 / 60 | 28.43 / 0.17 / 65 | 20.60 / 0.10 / 65 | 18.25 / 0.16 / 60 |
| 8 | 15.80 / 0.43 / 50 | 10.20 / 0.36 / 59 | 10.70 / 0.38 / 71 | 15.80 / 0.22 / 50 | 10.20 / 0.36 / 59 | 10.90 / 0.11 / 71 |
| 9 | 21.20 / 0.85 / 82 | 10.00 / 0.38 / 80 | 9.00 / 0.47 / 90 | 20.80 / 0.39 / 82 | 10.80 / 0.26 / 80 | 10.20 / 0.79 / 90 |
| 10 | 19.95 / 2.30 / 58 | 13.00 / 1.40 / 58 | - / - / 100 | 19.78 / 2.48 / 58 | 13.50 / 0.30 / 58 | - / - / 100 |
| 11 | 21.98 / 0.23 / 40 | 11.25 / 0.26 / 42 | 16.70 / 0.48 / 45 | - / - / 40 | - / - / 42 | - / - / 45 |
| 12 | 21.48 / 0.47 / 85 | 14.55 / 0.43 / 85 | 17.18 / 0.12 /85 | 20.80 / 0.22 / 85 | 13.68 / 0.13 / 85 | 16.93 / 0.73 / 85 |

FDI, first dorsal interosseous muscle; BB, biceps brachii muscle; msec, milliseconds; mV, millivolts; “ – ” , not evoked.
